# The Exact Definition of the Boundary between the Rapid and Slow Ejection Phases on ECGs and Accurate Location of the j Point

**DOI:** 10.17691/stm2020.12.3.01

**Published:** 2020-06-28

**Authors:** O.K. Voronova, V.A. Zernov, M.Yu. Rudenko

**Affiliations:** Senior Researcher; Russian New University, 22 Radio St., Moscow, 105005, Russia; Professor, Rector; Russian New University, 22 Radio St., Moscow, 105005, Russia; Head of the Laboratory for Cardiovascular System Investigations; Russian New University, 22 Radio St., Moscow, 105005, Russia

**Keywords:** cardiometry, cardiology, hemodynamics, ECG, rheography, j point.

## Abstract

**The aim of the study** was to describe the boundary between the phases of rapid and slow ejection in the cardiac cycle and to define the exact location of the j point on ECGs.

**Materials and Methods.:**

The work is based on the theory of heart cycle phase analysis using mathematical equations of hemodynamics. The balance of phase diastolic and systolic blood volumes depending on the duration of the cardiac cycle phases has been verified by these equations. The interrelation of phase duration and phase blood volumes was employed to exactly define the cardiac cycle boundaries on ECGs. Synchronous recording of the ECG and rheogram was used to determine the precise location of the j point.

**Results.:**

The exact boundary between the phases of rapid and slow ejection has been defined. A new point L determining the boundary between SL–Lj phases was introduced for its designation.

**Conclusion.:**

The j point was previously considered to be part of an ECG depending on the body temperature and which does not always appear. It had an ambiguous definition: Osborn wave or j wave. At the same time, the precise boundary between the rapid and slow ejection phases, the blood volumes of which in the total amount are equal to the blood stroke volume, has not been identified exactly. The work performed allows for accurate definition of criteria for recording rapid and slow ejection phases and j point location on the ECG.

## Introduction

In classical electrocardiography, an important place is occupied by an ECG interpretation based on the analysis of intervals, segments, and waves [1, 2]. Though this analysis has been used for a long time, no well-defined criteria of defining their beginning and end have been established so far. Therefore, understanding of the boundaries of the systolic part of the ECG cardiac cycle structure has been formed from multiple contradictions. This is also referred to as the ST segment, the structure of which is poorly studied. An ambiguous and non-periodic change of its shape on the recorded ECGs did not provide the possibilities for studying it in detail. To compensate for the knowledge, high-resolution electrocardiography was proposed [3], however, it failed to achieve the goal and the ST segment remained *terra incognita* in the perception of the investigators.

The position of j point on the ECG became a subject of much controversy. Criteria variability for recording its location has transformed gradually into the suggestion to call this point a j wave which can appear on the ECG immediately following the QRS complex [4, 5] though some researchers went on to consider it to be positioned closer to the T wave [6]. The change in the body temperature was thought to be a clinical cause of appearing the j point [7].

The fact that the ST segment occupies only half the systole in the cardiac cycle makes us think that there is a lot to be explored in electrocardiography. This is confirmed by other authors as well: for example, they suggest the existence of some “invisible zones” [8].

At the beginning of the 2000s, publications on the heart cycle phase analysis appeared in Russia [9]. Two PhD theses were defended on this topic [10, 11]. In order to define more completely the biological processes pertaining to the systolic part of the ECG, the authors of the present article had to introduce new concepts into electrocardiography which allowed them to demarcate clearly the phase structure of the systolic part of the cardiac cycle.

**The aim of the study** was to create criteria for recording cardiac cycle phases in the ST segment on the ECG and to define exactly the location of the j point.

## Materials and Methods

A theory of the heart cycle phase analysis proposed previously by us and hemodynamic equations by G. Poyedintsev and O. Voronova were used in our work [9]. These equations are based on the indirect methods of calculating phase blood volumes. Durations of the cardiac cycle phases serve as functions for the calculation of volume characteristics. A phase structure of the ST segment consists of the phases of tension, rapid ejection, and slow ejection. Considering the fact that previously there were no accurate criteria for recording the boundaries of these phases, the principle of comparing the known and unknown was employed. Under this condition, the selected equations of hemodynamics allow the calculation of systolic and diastolic phase blood volumes.

The boundaries of the diastolic phases of the cardiac cycle are fixed clearly on the ECG. The criteria for recording the phases of early atrial diastole and systole in electrocardiography do not raise doubts. The equality is based on the law of conservation of energy: the amount of blood which enters and leaves the heart must be equal. Therefore, composing the equation from the sum of systolic and diastolic phase volumes, the causes of the equality divergences can be seen and eliminated.

This equation is presented as:

*PV*_1_+*PV*_2_=*PV*_3_+*PV*_4_,

where *PV*_1_ is the blood volume flowing into the ventricle during the early diastole (ml); *PV*_2_ is the blood volume flowing into the ventricle during the atrial systole (ml); *PV*_3_ is the blood volume ejected from the ventricle during rapid ejection (ml); *PV*_4_ is the blood volume ejected from the ventricle during slow ejection (ml).

In *the first stage*, it was necessary to accurately define the criterion for recording the S point, i.e. the beginning of the tension phase. The calculation data obtained in the course of studying real ECGs showed that the S point is located at the point of bending of the right part of the S wave. It is in line with the logic of the biophysical processes forming the ECG contour [9]. Using the mathematical first derivative of differential calculus of the wave, S may be easily fixed in 100% of real ECGs. As the result, we succeeded in achieving the equality of systolic and diastolic blood volumes. This fact signifies the correctness of establishing the recording criteria for the S point.

Then, it was necessary to obtain separately components of the systolic blood volume, i.e. *PV*_3_+*PV*_4_. For this purpose, it was required to establish the criterion for fixing the demarcation of rapid and slow ejection phases. Keeping in mind the fact that there is also the tension phase in the ST segment, it should be taken into consideration as well. During this phase, the aortic valve is closed and blood does not flow into aorta.

**Figure 1:**
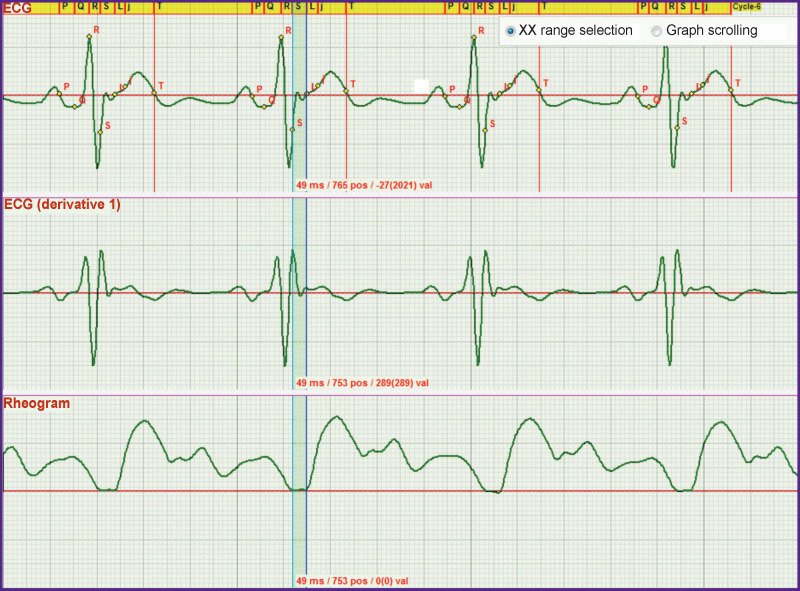
Synchronously recorded ECG and rheogra Beginning of the aortic valve opening corresponds to the beginning of the rapid ejection phase. To indicate this moment on the ECG, a new designation, the L point (one of the bend points), was introduced

In *the second stage*, a more difficult task that was to solved consisted in establishing the criteria for fixing the moment when the aortic valve begins to open. A synchronous recording of the ECG and rheogram was used to achieve the goal (Figure 1).

As seen in Figure 1, a small wave on the ECG corresponds to the moment of the beginning of the rheogram curve rise. The investigations have shown that this wave appears in each recorded ECG. Taking into account that there is no description of the criteria for its fixation in the literature and understanding its importance for functioning of the heart, we decided to designate this point by the letter L. Thus, the L point is the onset of the rapid ejection phase.

**Figure 2:**
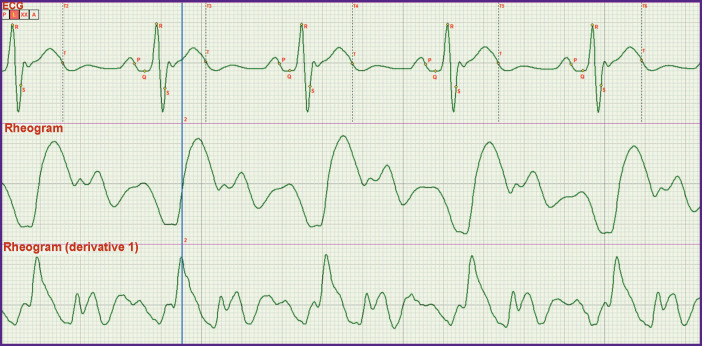
Synchronously recorded ECG and rheogram

In *the third stage*, it was necessary to specify more exactly the criteria for fixing the end of the rapid ejection phase which is at the same time the beginning of the slow ejection phase. Actually, it meant the precise determination of the j point location. And again, the synchronous recording of the ECG and rheogram was performed (Figure 2).

The j point corresponds to the bend point of the ascending part of the reogram curve and is determined by the maximum of the first derivative of the rheogram. This corresponds to the end of the second small ECG wave before the T point

Figure 2 shows that the point of the curve bending of the rheogram ascending part is clearly defined by the maximum of the first derivative. However, this moment corresponds to the end of the small wave on the ECG locating before the T wave. This is just the position of the j point. In the literature, its location is indicated only approximately. Putting phase durations SL and Lj in the hemodynamic equation by G. Poyedintsev and O. Voronova we obtained phase systolic blood volumes, the sum of which was equal to that of diastolic volumes.

Further investigations fully confirmed that the employment of the criteria for fixing SL and Lj phases is true to fact and the abovementioned equation holds with high precision in 100% of cases.

## Results and Discussion

The main result of the present work was the establishment of criteria for recording the boundary between the phases of rapid and slow ejection in the phase structure of the cardiac cycle. The second important result was an exact description of the j point location.

The work reflects the possibilities of classic scientific approaches to the research practice. It appeared that one channel is quite sufficient for ECG recording. It is important that this channel records a signal in aorta, the processes in which reflect the entire activity of the cardiovascular system [9]. Besides, it was important to compare the phase correlations of an ECG with aortic rheogram.

**Figure 3:**
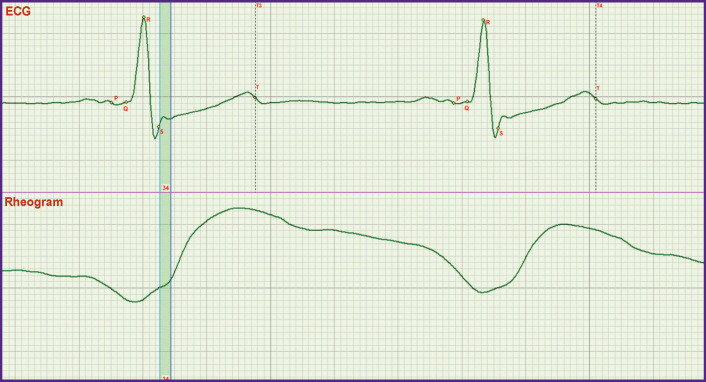
The possibility of qualitatively assess the alterations of filling the aorta with blood when the aortic valve is closed Rise of the rheogram curve up to the S point indicates that blood volumes enter the aorta through the closed aortic valve

Previously [9], an ECG and rheogram recording has also been used but it was a multichannel lead ECGs and rheography of the chest which covers the processes of filling all the chest organs with blood. Not knowing the exact location of one of the key points S, synchronization of the rheogram was performed by fixing its minimum to the isoline. In the given work, the rheogram isoline was fixed at the point corresponding to the S point [9]. The exact definition of the S point position made it possible to analyze the mechanism of regulation of the diastolic arterial pressure by means of rheography. It was assessed by the presence or absence of blood filling according to the rheogram curve up to the S point (Figure 3). Previously, it could not be done.

In Figure 3, the process of the rheogram curve rising up to the S point is presented. Comparison of Figures 1 and 2 shows that in this case penetration of blood through the closed aortic valve is observed. If the rheogram had been fixed by its minimum to the isoline, it would be difficult to assess the presence of this rise which is the criterion for serious alterations in the work of the aortic valve.

**Figure 4:**
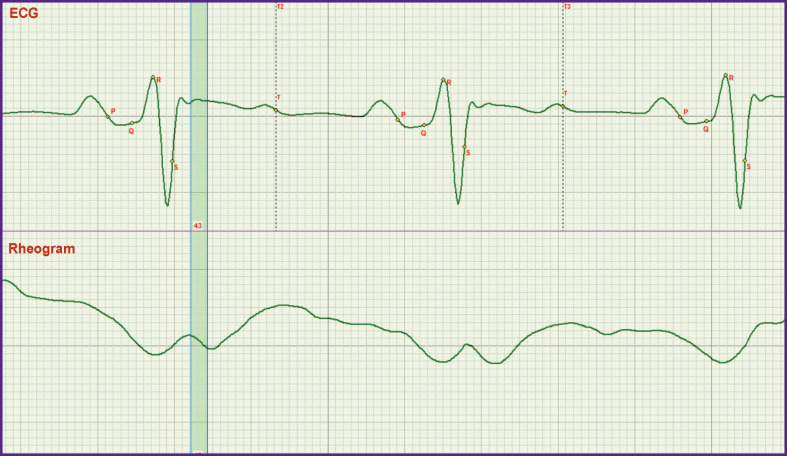
Absence of the normal development of the rapid ejection phase The curve of the rheogram has a negative slope in the marked rapid ejection phase

New possibilities are also opened in the evaluation of the rapid and slow ejection phases. It is seen in Figure 4 that growth of rheogram in Lj phase is absent. This has important clinical consequences: it signifies the impossibility of forming the blood flow structure possessing the properties of increased fluidity [9]. It may result in formation of thrombi in large bloodvessels.

**Figure 5:**
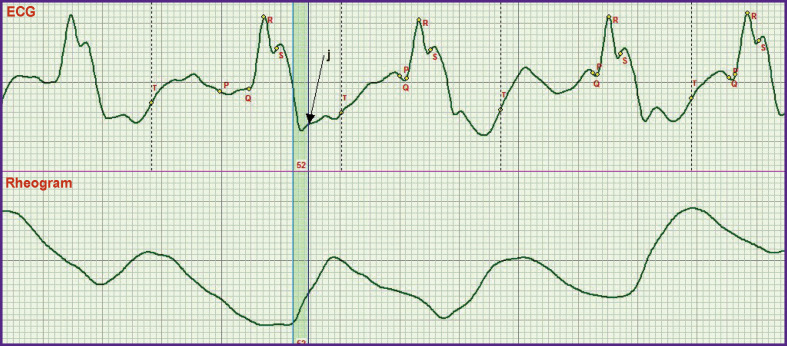
The location of the j point corresponds to the end of the rapid ejection phase marked in the figure by arrow (this is verified by the correspondence to the middle of the rheogram rising edge)

The definition of the Osborn wave may be seen in a new light. The ECG record corresponding to this wave is presented in Figure 5. However, it is necessary to distinguish the meanings of the words “wave” and “point”. The j point corresponds to the end of the rapid ejection phase marked in Figure 5 with a vertical strip. This is the middle of the rheogram curve rise, whereas the Osborn wave is on the left from the phase.

## Conclusion

The heart cycle phase analysis based on the mathematical models of hemodynamics suggested by G. Poyedintsev and O. Voronova provided the opportunity to remove a veil of the unknown from the ECG which concealed the structure of the three systolic phases of the cardiac cycle: tension, rapid, and slow ejection. No doubts that the results of the work will allow the researchers to supplement and clarify the existing concepts in electrocardiography [12–18].

Understanding the mechanism of formation of these phases will give cardiologists the possibility to have a key to the exploration of unknown mechanisms of the cardiovascular system functioning which still keeps lots of secrets. Their solution will bring us closer to the creation of an artificial heart.
